# Translation of PET radiotracers for cancer imaging: recommendations from the National Cancer Imaging Translational Accelerator (NCITA) consensus meeting

**DOI:** 10.1186/s12916-024-03831-z

**Published:** 2025-01-23

**Authors:** Martina A. McAteer, Daniel R. McGowan, Gary J. R. Cook, Hing Y. Leung, Tony Ng, James P. B. O’Connor, Luigi Aloj, Anna Barnes, Phil J. Blower, Kevin M. Brindle, John Braun, Craig Buckley, Daniel Darian, Paul Evans, Vicky Goh, David Grainger, Carol Green, Matt G. Hall, Thomas A. Harding, Catherine D. G. Hines, Simon J. Hollingsworth, Penny L. Hubbard Cristinacce, Rowland O. Illing, Martin Lee, Baptiste Leurent, Sue Mallett, Radhouene Neji, Natalia Norori, Nora Pashayan, Neel Patel, Kieran Prior, Thomas Reiner, Adam Retter, Alasdair Taylor, Jasper van der Aart, Joseph Woollcott, Wai-Lup Wong, Jan van der Meulen, Shonit Punwani, Geoff S. Higgins

**Affiliations:** 1 Department of Oncology, University of Oxford, Oxford, UK; 2 Department of Medical Physics and Clinical Engineering, Oxford University Hospitals NHS Foundation Trust, Oxford, UK; 3 School of Biomedical Engineering and Imaging Sciences, King’s College London, London, UK; 4 King’s College London and Guy’s and St Thomas’ PET Centre, St Thomas’ Hospital, London, UK; 5 CRUK Scotland Institute, Glasgow, UK; 6 School of Cancer Sciences, University of Glasgow, Glasgow, UK; 7 School of Cancer & Pharmaceutical Sciences, King’s College London, London, UK; 8 Oncology Translational Research, GSK, Stevenage, UK; 9 Division of Cancer Sciences, University of Manchester, Manchester, UK; 10 Division of Radiotherapy and Imaging, Institute of Cancer Research, London, UK; 11 Department of Radiology, University of Cambridge, Cambridge, UK; 12 Southeast Region, Office of the Chief Scientific Officer, NHS-England, England, UK; 13 King’s Technology Evaluation Centre (KiTEC), School of Biomedical Engineering & Imaging Science, King’s College London, London, UK; 14 Cancer Research UK Cambridge Institute, University of Cambridge, Cambridge, UK; 15 RMH Radiotherapy Focus Group & RMH Biomedical Research Centre Consumer Group, Sutton, UK; 16 Siemens Healthcare Limited, Camberley, UK; 17 GE HealthCare, Pharmaceutical Diagnostics, Chalfont St. Giles, UK; 18 Department of Radiology, NHS Foundation Trust, Guy’s and St Thomas, London, UK; 19 Medicines and Healthcare Products Regulatory Agency, London, UK; 20 Patient and Public Representative, Oxford, UK; 21 National Physical Laboratory, Teddington, UK; 22 Prostate Cancer UK, London, UK; 23 Population Health Sciences, University of Bristol, Bristol, UK; 24 GSK, Collegeville, PA USA; 25 Late Development Oncology, AstraZeneca, Cambridge, UK; 26 Department of Surgery & Interventional Science, University College London, London, UK; 27 Clinical Trial and Statistics Unit, Institute of Cancer Research, Sutton, UK; 28 The Royal Marsden Clinical Research Facility, London, UK; 29 Department of Statistical Science, University College London, London, UK; 30 Centre for Medical Imaging, University College London, London, UK; 31 Department of Applied Health Research, University College London, London, UK; 32 Department of Public Health and Primary Care, University of Cambridge, Cambridge, UK; 33 Department of Radiology, Churchill Hospital, Oxford University NHS Foundation Trust, Oxford, UK; 34 Telix Pharmaceuticals Limited, North Melbourne, Australia; 35 Cancer Research UK, London, UK; 36 Evergreen Theragnostics, Springfield, NJ 07081 USA; 37 University Hospitals of Morecambe Bay NHS Foundation Trust, Royal Lancaster Infirmary, Lancaster, UK; 38 GSK Research and Development, Hertfordshire, UK; 39 PET CT Department, Strickland Scanner Centre Mount Vernon Hospital, Northwood, UK; 40 Department of Health Services Research & Policy, London School of Hygiene & Tropical Medicine, London, UK

**Keywords:** Consensus guidelines, Positron emission tomography, Cancer imaging, Preclinical evaluation, Clinical validation, Translational methods, Radiotracers, Diagnostic imaging, Imaging biomarker, RAND/UCLA Appropriateness Method

## Abstract

**Background:**

The clinical translation of positron emission tomography (PET) radiotracers for cancer management presents complex challenges. We have developed consensus-based recommendations for preclinical and clinical assessment of novel and established radiotracers, applied to image different cancer types, to improve the standardisation of translational methodologies and accelerate clinical implementation.

**Methods:**

A consensus process was developed using the RAND/UCLA Appropriateness Method (RAM) to gather insights from a multidisciplinary panel of 38 key stakeholders on the appropriateness of preclinical and clinical methodologies and stakeholder engagement for PET radiotracer translation. Panellists independently completed a consensus survey of 57 questions, rating each on a 9-point Likert scale. Subsequently, panellists attended a consensus meeting to discuss survey outcomes and readjust scores independently if desired. Survey items with median scores ≥ 7 were considered ‘required/appropriate’, ≤ 3 ‘not required/inappropriate’, and 4–6 indicated ‘uncertainty remained’. Consensus was determined as ~ 70% participant agreement on whether the item was ‘required/appropriate’ or ‘not required/not appropriate’.

**Results:**

Consensus was achieved for 38 of 57 (67%) survey questions related to preclinical and clinical methodologies, and stakeholder engagement. For evaluating established radiotracers in new cancer types, in vitro and preclinical studies were considered unnecessary, clinical pharmacokinetic studies were considered appropriate, and clinical dosimetry and biodistribution studies were considered unnecessary, if sufficient previous data existed. There was ‘agreement without consensus’ that clinical repeatability and reproducibility studies are required while ‘uncertainty remained’ regarding the need for comparison studies. For novel radiotracers, in vitro and preclinical studies, such as dosimetry and/or biodistribution studies and tumour histological assessment were considered appropriate, as well as comprehensive clinical validation. Conversely, preclinical reproducibility studies were considered unnecessary and ‘uncertainties remained’ regarding preclinical pharmacokinetic and repeatability evaluation. Other consensus areas included standardisation of clinical study protocols, streamlined regulatory frameworks and patient and public involvement. While a centralised UK clinical imaging research infrastructure and open access federated data repository were considered necessary, there was ‘agreement without consensus’ regarding the requirement for a centralised UK preclinical imaging infrastructure.

**Conclusions:**

We provide consensus-based recommendations, emphasising streamlined methodologies and regulatory frameworks, together with active stakeholder engagement, for improving PET radiotracer standardisation, reproducibility and clinical implementation in oncology.

**Supplementary Information:**

The online version contains supplementary material available at 10.1186/s12916-024-03831-z.

## Background

Positron emission tomography (PET) is a powerful, minimally invasive imaging modality for visualising and quantifying molecular targets and processes in tumours using specific radiotracers [1]. The combination of PET with computed tomography (PET/CT), or more recently, magnetic resonance imaging (PET/MRI), has the capacity to transform clinical medicine, providing functional and anatomical information for tumour detection and treatment response monitoring. In oncology, the most widely used radiotracer is the glucose analogue [^18^F]fluoro-2-deoxy-d-glucose ([^18^F]FDG), approved by the U.S. Food and Drug Administration (FDA) for oncological indications [2], which leverages the Warburg effect. However, its specificity is limited [3] by accumulation in noncancerous tissues with high glucose metabolism, due to infection or inflammatory processes [4]. Moreover, while H^18^F]FDG is taken up into cells via the facilitated glucose transporters it is not a substrate for sodium-dependent glucose transporters and therefore there can be a disconnect between [^18^F]FDG uptake and glucose utilisation [5].

PET/CT and PET/MRI methods have a rapidly expanding and diverse portfolio of radiotracers. Receptor targeting radiotracers, such as [^68^ Ga]-Ga-DOTA-TATE, targeting somatostatin receptor 2 expression in neuroendocrine [6] and thymic endothelial tumours [7], [^68^ Ga]-Ga-PSMA (prostate-specific membrane antigen) [8] and [^18^F]F-PSMA [9], primarily targeting PSMA expression in prostate cancer, and quinoline-based [^68^ Ga]-labelled tracers that target fibroblast-activation-protein overexpressed in primary and metastatic tumours [10–12] have emerged as potent alternatives to [^18^F]FDG for cancer detection and monitoring. The development of radiolabelled amino acid-based analogues that detect aberrantly upregulated amino acid metabolism [13], offers adaptable imaging for various cancers and neurological conditions, without relying on specific receptor expression. For example, FDA-approved [^18^F]3,4-dihydroxy-6-fluoro-L-phenylalanine ([^18^F]FDOPA) for PET imaging of Parkinson’s disease and related syndromes [14, 15] shows promise in schizophrenia [16] and brain tumour detection [17]. Additionally, [^18^F]FDOPA, [^18^F]fluoroethyltyrosine and [^11^C]methionine are used for glioma detection and grading [18, 19], complementing MRI in recurrent glioma management [20, 21] and outperforming [^18^F]FDG PET/CT in distinguishing high- and low-grade gliomas [22, 23]. [^18^F]fluciclovine (Axumin®), FDA-approved for PET imaging of prostate cancer [24], also demonstrates potential for PET detection of recurrent brain tumours [25], multiple myeloma [26] and PET/MRI of brain metastases [27].

Despite promising advances in PET imaging technologies, such as clinical PET/MRI [28] and total body PET [29], the development of artificial intelligence (AI) tools [30] and ongoing efforts to standardise translational methodologies [31–33] and protocols [2, 34, 35], the clinical implementation of novel or established radiotracers for cancer diagnosis and treatment decision-making remains limited. This challenge is compounded by the complexities of the translational pipeline, including different study methodologies for preclinical and clinical assessment of radiotracers, variations in image acquisition, processing, data analysis and reporting across different centres and scanner vendors [22, 36, 37], introducing significant heterogeneity that hampers reproducibility of study findings. Stringent regulatory requirements can further impede clinical adoption. For example, in the UK, PET radiotracers, unlike other diagnostics, must undergo a central commissioning process by the National Health Service (NHS) England, competing with other specialised services, predominantly therapeutics, leading to prolonged delays. Evidence of clinical- and cost-effectiveness is necessary before commissioning is considered [38, 39] and marketing authorisation is usually required before PET radiotracers can be sold, supplied or exported. To address these challenges, the British Nuclear Medicine Society (BNMS) PET-CT Commissioning Manifesto have proposed prioritising clinical need over marketing authorisation, emphasising evidence of incremental clinical and/or economic benefits compared to standard management, focusing on improvements in radiotracer diagnostic metrics, rather than survival outcomes [39].

Here, we present the findings of a collaborative consensus process, involving a multidisciplinary panel, including stakeholders from academia, industry, UK funding and regulatory bodies, NHS England and patient and public involvement (PPI). Our objective was to review translational methodologies for evaluating novel and existing PET radiotracers in preclinical and clinical cancer imaging studies, to identify areas of uncertainty, and formulate consensus recommendations for streamlining radiotracer development and reproducibility, to accelerate clinical adoption of PET radiotracers for cancer imaging. While the integration of radiotracers into the NHS serves as a specific example of the complexities involved in clinical adoption, our consensus recommendations and methodologies discussed are intended to be broadly applicable for the international research community, which faces similar challenges in standardising practices across the radiotracer translational pipeline and navigating regulatory requirements.

## Methods

The National Cancer Imaging Translational Accelerator (NCITA)—a UK-based clinical imaging research infrastructure dedicated to accelerating the validation and translation of imaging biomarkers into routine clinical practice [40]—convened a remote questionnaire-based survey and hybrid consensus panel discussion on PET radiotracer development and translation practices.

### Consensus method

The RAND/UCLA Appropriateness Method (RAM) was selected to develop a consensus strategy due to its suitability for areas lacking in sufficient evidence applicable to diverse patient populations in clinical practice or with insufficient level 1 evidence (such as systematic reviews) [41].

The RAM user’s manual [42] guided the consensus process, which combined scientific evidence with the collective opinions of a diverse, multidisciplinary panel, obtained using two rounds of surveys and a consensus meeting.

The panel comprised of 38 voting members with expertise and experience in translational imaging and oncology, including medical imaging experts, clinical oncologists, physicists, and research scientists from leading UK academic institutions, NHS Foundation Trust Hospitals, and industry representatives from the UK, USA and Australia. The panel also included stakeholder representatives from NHS England, UK funding organisations, regulatory bodies, as well as individuals advocating for patient and public involvement (PPI) in research (Additional file 1: Table S1).

### Round 1: remote survey

The first-round survey questions were developed based on a combination of a literature review, an informal pilot questionnaire circulated to academic and industry collaborators within the NCITA network, and authors of the Imaging Roadmap paper [31] as well as an internal review of the challenges in the translational pipeline for the NCITA Exemplar projects [43] and a discussion on the collective feedback by MAMcA, DRMcG, GSH, SP and AR. Panellists participated in the first-round survey, consisting of 57 questions and six optional free-text fields at the end of each survey section, using the JISC Online Survey platform (https://www.onlinesurveys.ac.uk/). The survey topics focused on preclinical and clinical methodologies for assessing novel and established PET radiotracers, applied to image new cancer types, and stakeholder engagement across the translational pipeline.

Participants individually rated the appropriateness of survey topics using a 9-point Likert scale, ranging from 1 (not required/ inappropriate) to 9 (required/ appropriate) and were given the option to select ‘Do not know’ if they did not have sufficient experience to answer a question.

### Data analysis

Survey results were assessed according to the RAM user’s manual [44], with median scores calculated for each survey item and grouped into a 3-point median score range of 1–3 (indicating not required/ inappropriate), 7–9 (indicating required/ appropriate) or 4–6 (indicating that uncertainty remained). Only questions scored by at least 53% of participants were considered for consensus, as described previously [45]. Consensus on the inappropriateness or appropriateness of a survey item was determined by evaluating the number of participant scores falling outside the median range 1–3 or 7–9, respectively, in relation to the number of participant responses per question, as shown in Table 1. This methodology was derived and extrapolated from Table 4 of the RAM user’s manual [44] as described previously [46]. This translated to ≥ 70% ‘agreement with consensus’ for questions answered by 20–25 participants or ≥ 69% for questions answered by at least 26 participants. There was ‘agreement without consensus’, when fewer scores fell within the median 3-point score ranges 1–3 or 7–9 and for survey items with a median score range of 4–6, ‘uncertainty remained’ regarding their appropriateness.

**Table 1:** Criteria for determining consensus for each survey question

Number of participant responses to a survey question	Number of participant responses OUTSIDE the median score range	% median scores INSIDE median score range for consensus
8–10	No more than 2	75–80
11–13	No more than 3	73–77
14–16	No more than 4	71–75
17–19	No more than 5	71–74
20–22	No more than 6	70–73
23–25	No more than 7	70–72
26–28	No more than 8	69–71
29–31	No more than 9	69–71
32–34	No more than 10	69–71
35–37	No more than 11	69–70

Consensus on the appropriateness or inappropriateness of a survey topic was reached if the number of participant responses outside the median 3-point score range (1–3 or 7–9, respectively) were within the limits defined in Table 1. If the number of participant responses outside the median score ranges 1–3 or 7–9 exceeded the limits in the table, then there was ‘agreement without consensus’ that the survey item is not required/ inappropriate or required/ appropriate. If the median score for a survey item was in the 4–6 median score range, then uncertainty remains regarding its requirement

### Round 2: consensus meeting and re-scoring of survey

**Fig. 1:**
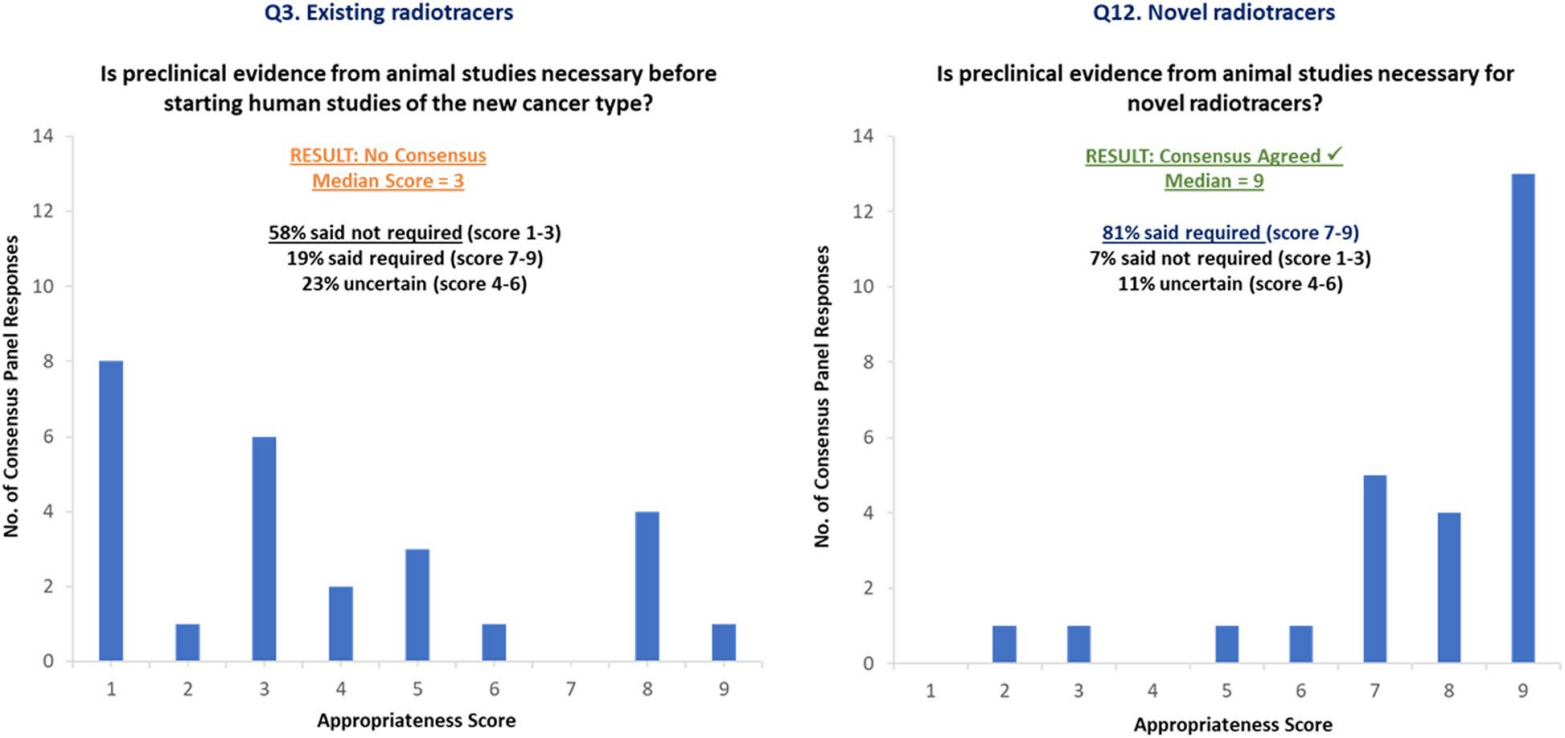
Examples of bar charts displayed to the consensus panel during the consensus meeting

A second round was held, consisting of a consensus meeting where participants reviewed the collective results from the Round 1 survey, and had the opportunity to adjust their ratings based on the discussion. This approach, recommended by the RAM consensus method, enabled improved decision quality and ensured that the consensus recommendations were generated from a well-rounded deliberation, rather than solely relying on the initial opinions of individual panel members.

The consensus meeting was held on 23 June 2023 in London, moderated by an independent chairperson (JvdM), with expertise in consensus methodology. At the beginning of the meeting, participants received printed copies of their individual scores from the first-round survey. Distribution bar charts representing the median scores for each survey question were presented (Fig. 1) followed by an open discussion, led by the independent chair, to review and clarify each question and its median score result. If necessary, a question could be rephrased to improve clarity, with consensus panel agreement, before rescoring. After the discussion for each question, panellists were asked to independently rescore their responses to the question on the printed copy of their individual first-round survey scores, with flexibility to maintain or change their original score. Panellists also had the option to select ‘Do Not Know’, which would exclude their response from the reanalysed distribution scores for that question. Panellists could also provide any further comments in the free-text boxes at the end of each survey section.

The meeting audio recording, summary notes and updated survey link with reworded questions were shared with panellists who could not attend the consensus meeting to enable remote participation in survey rescoring. The second-round rescoring data were analysed as described above.

## Results

The first-round online survey received 32 out of 34 responses (94%) prior to the consensus meeting. This included two joint responses from panellists at Prostate Cancer UK (*n* = 3) and Siemens Healthineers (*n* = 3). The consensus meeting was attended by 30 panellists, either in-person (*n* = 23) or virtually (*n* = 7). Of those who were not able to attend the meeting, three remotely rescored their survey responses after the meeting; two delegated rescoring to attendees from their institution and three did not participate. Therefore, considering that two members delegated rescoring, the second-round rescoring of the survey was completed by 33 out of 36 panellists (92%). The minimum number of participant responses received for any survey question was 25 out of 32 responses (78%) in the first-round survey, and 25 out of 33 responses (76%) in the second-round survey. Therefore, all survey questions passed the response rate threshold for consideration for consensus i.e. at least 53% participant response rate [45].

During the consensus meeting, 15 survey questions were amended to improve clarity, with amendments highlighted in bold in Additional file 1: Tables S2 and S3. No questions were excluded. The word ‘required’ in the questions was clarified during the consensus discussion to refer to what steps are appropriate for the successful translation of PET radiotracers along the translational pipeline into routine clinical practice. The median response scores, appropriateness/requirement, consensus levels and number of abstentions for each question in the second-round survey are summarised in Additional file 1: Tables S2 and S3, and first-round survey results are summarised in Additional file 1: Tables S4 and S5. All questions passed the minimum threshold of at least 53% participant responses. Overall, consensus was reached on 38 of 57 survey questions (67%) after the second-round survey compared to 24 of 57 questions (42%) after the first-round survey (prior to the consensus meeting).

Consensus was reached for 6 of 8 questions (75%) on preclinical evaluation of an established PET radiotracer in a new cancer type, indicating that in vitro and in vivo preclinical studies are typically not required before clinical evaluation. However, for clinical evaluation of established radiotracers, only 2 out of 9 questions (22%) reached consensus, with agreement that clinical pharmacokinetic studies are required while dosimetry and/or biodistribution studies are not.

For novel radiotracers, consensus was reached on 5 of 7 questions (71%) for preclinical evaluation, emphasising the requirement for in vitro and in vivo preclinical evidence, such as dosimetry and/or biodistribution and histological evaluation, while preclinical reproducibility studies are not required. For clinical evaluation of novel radiotracers, consensus was achieved for 7 of 9 questions (78%), including the requirement for clinical scanner phantom studies, dosimetry and/or biodistribution, pharmacokinetic studies, repeatability and reproducibility studies.

Consensus was reached for 2 of 7 questions (29%) on stakeholder involvement in the preclinical phase, emphasising the role of funders in encouraging and offering guidance on PPI. During the clinical phase, consensus was reached for 16 of 18 questions (89%), highlighting the importance of harmonised protocols, quality management, PPI involvement, utilisation of a PET core lab, open access federated repository and a centralised infrastructure, like NCITA. Consensus also supported the need for a regulatory framework outlining requirements for diagnostic, prognostic and therapy assessment radiotracers, and guidelines for clinical implementation of established PET radiotracers for imaging a different cancer type. Comparative studies were deemed necessary for health economics evaluation prior to clinical implementation of novel or repurposed radiotracers at a national level, along with enhanced collaboration with international research efforts, encouraged by funders, and a gap analysis on UK PET imaging workforce issues and training requirements.

The panel formed consensus-based recommendations on these areas of ‘agreement with consensus’ for preclinical and clinical studies using established or novel PET radiotracers for cancer imaging, as outlined in Tables 2 and 3, respectively. Additional comments provided in the six optional free-text fields at the end of each survey section are summarised in Additional file 1: Tables S6, S7 and S8).

**Table 2:** Consensus recommendations for preclinical studies using established or novel radiotracers

Consensus recommendations for preclinical PET radiotracer evaluation
• When sufficient clinical pharmacological data exists for an established PET radiotracer, additional in vitro and in vivo preclinical studies are not necessary for clinical application in a new indication/new cancer type
• To assess a novel PET radiotracer for cancer imaging, it is appropriate to conduct in vitro studies and to obtain in vivo preclinical evidence to assess organ biodistribution and associated radiation dosimetry
• Where feasible, preclinical histological assessment is required to confirm binding of a novel PET radiotracer to the tumour target
• Preclinical, multicentre reproducibility studies are not required for new or existing PET radiotracers as single-centre studies with appropriate statistical power are deemed sufficient
• It is recommended that funding bodies offer comprehensive guidance to facilitate meaningful PPI engagement throughout the preclinical study process

The consensus panel developed recommendations based on survey topics that achieved ‘agreement with consensus’ for preclinical studies using established or novel PET radiotracers

**Table 3:** Consensus recommendations for clinical translation of established or novel PET radiotracers

Consensus recommendations for clinical translation of PET radiotracers
• Phantom studies are required to calibrate clinical scanners and validate imaging protocols using novel PET radiotracers
• Clinical dosimetry and/or biodistribution studies are required for novel PET radiotracer imaging studies in cancer
• It is not necessary to conduct clinical dosimetry and biodistribution studies for established radiotracers, when sufficient previous data are available for a different indication
• Clinical pharmacokinetic studies using an established PET radiotracer are required to assess sensitivity for detecting a new tumour type and/or to optimise timing of use
• Clinical pharmacokinetic studies with novel PET radiotracers are required to assess sensitivity and/or optimise timing of use for cancer imaging
• Repeatability and reproducibility studies are required to validate new PET radiotracers for cancer diagnosis, prognosis or prediction of treatment responses
• Healthy volunteers are not required for multicentre validation studies
• Harmonised study protocols and quality management are required for multicentre clinical validation studies
• Involvement of a centralised PET core lab is recommended to support multicentre clinical validation radiotracer studies
• A regulatory framework is required to standardise PET radiotracer development for cancer imaging and facilitate their translation into clinical practice
• The proposed regulatory framework should mandate researchers to seek expert statistical advice prior to conducting preclinical and clinical PET radiotracer studies to ensure robust statistical design and accurate sample size determination of the study protocols
• The proposed regulatory framework should delineate study requirements for diagnostic, prognostic and therapeutic response PET radiotracer evaluation
• It is recommended that the proposed regulatory framework integrates guidelines for Research Ethics Committees (REC) to ensure harmonised standards for evaluating clinical, technical and biological validation evidence for cancer imaging radiotracers
• Health Technology Assessment (HTA) bodies, such as NICE, should offer guidelines for integrating new and established PET radiotracers for cancer imaging into clinical practice
• A comparison study to the standard of care imaging technique is required for health economics evaluation of cancer PET imaging radiotracers prior to their adoption into clinical practice at a national level
• Researchers are strongly recommended to involve PPI from the initial planning and development stages of their clinical PET radiotracer validation studies
• A centralised UK infrastructure, such as NCITA, is needed to accelerate clinical PET radiotracer validation for cancer imaging
• An open access federated repository is required to share, where possible, clinical data from repeatability and reproducibility PET imaging studies between industry and academia, emphasising adherence to FAIR principles; that data must be Findable, Accessible, Interoperable and Reusable
• A federated repository is required for AI algorithm development, enhancing workflows for clinical PET imaging data analysis
• Funders should incentivise greater collaboration between UK and international clinical imaging research initiatives to improve reproducibility in quantitative PET cancer imaging
• A gap analysis is needed to evaluate workforce and training requirements to accelerate the adoption of new PET radiotracers into clinical practice

The consensus panel developed recommendations based on survey topics that achieved ‘agreement with consensus’ for clinical studies using established or novel PET radiotracers

## Discussion

The following sections discuss areas of ‘agreement with consensus’ as well as areas of ‘agreement without consensus’ or ‘uncertainty’ (as defined in the Data Analysis section above) regarding the development and translation of novel and established PET radiotracers for cancer imaging.

### Preclinical *in vitro* and *in vivo* characterisation of an established PET radiotracer

There was ‘agreement with consensus’ that in vitro studies, such as radiotracer binding affinity, specificity, metabolism and stability, are not necessary for evaluating an established radiotracer for imaging a new cancer type, if robust human data are available in a different cancer type. There was ‘agreement without consensus’ that phantom studies are required, in addition to routine preclinical scanner calibration, to assess scanner performance and validate imaging acquisition and reconstruction protocols for an established radiotracer. The phantom studies may involve solid vessels of various sizes and geometry filled with different radiotracer concentrations, or three-dimensional phantoms that simulate radiotracer uptake in a specific organ [47, 48]. However, some panellists suggested that additional phantom studies are not necessary if the radiotracer has sufficient previous preclinical phantom study data.

There was consensus that preclinical dosimetry, biodistribution and pharmacokinetic studies are generally not required for applying an existing radiotracer to image a new cancer type. However, it was recognised that these studies may provide valuable insights for radiotracers with limited previous preclinical data. There was ‘agreement without consensus’ on the need for preclinical histological evaluation of the radiotracer's target specificity in the new tumour type. Some suggested that while previous histological evidence of radiotracer specificity in a different cancer type may be sufficient, the need for histology should be evaluated on a study-by-study basis.

While beyond the scope of this consensus discussion, published datasets of RNA sequencing of cancer cell lines, such as those available from the Broad Institute Cancer Cell Line Encyclopedia (CCLE) [49] in addition to proteomic datasets for cancers from e.g. The Human Protein Atlas [50], can serve as valuable resources for data-driven investigations, prior to commencing studies to apply an existing radiotracer to new cancer type.

### Preclinical *in vitro* and *in vivo* characterisation of a new/novel radiotracer

Consensus was reached that in vitro binding affinity, selectivity, stability and kinetic studies are appropriate for characterisation of the diagnostic accuracy of a new PET radiotracer. Despite differences in radiotracer concentrations, in vitro assessment has shown promise in predicting human radiotracer metabolism [20]. However, it was noted that some radiotracers may exhibit suboptimal in vivo kinetics and high non-specific binding, despite promising in vitro assessment, highlighting the importance of rapid progression to in vivo preclinical studies [51]. In vitro studies may assist in the design of subsequent in vivo experiments [52], minimising the number of animals, in line with the principles of the 3Rs (Replacement, Reduction, Refinement) [53].

Consensus was agreed that in vivo preclinical evidence is highly appropriate to assess organ biodistribution and associated radiation dosimetry for new PET radiotracers. The development of small animal total body PET scanners, which can simultaneously image all areas of the body with equal sensitivity, offers the potential to improve quantitative accuracy in preclinical PET imaging studies [54]. However, radiotracer biodistribution may vary due to differences in metabolic rates and the differences in organs between preclinical animal models and humans [55].

It was agreed by consensus that while preclinical dosimetry studies are required for novel radiotracers, it was acknowledged that these studies may not always predict clinical dosimetry profiles [55]. Some also questioned the utility of preclinical dosimetry, given that first-in-human studies typically include dosimetry assessment [51]. There was also uncertainty regarding the appropriateness of preclinical pharmacokinetic studies, due to the influence of factors such as anaesthesia and animal handling [56] on radiotracer pharmacokinetics and clearance. Furthermore, partial volume effects in small organs [57, 58] may decrease the accuracy of radiotracer uptake measurements. However, despite physiological differences, preclinical studies can offer reasonable estimates of expected pharmacokinetic and toxicological profiles in humans [52].

The panel reached consensus on the need for histological correlation to assess a novel radiotracer’s specificity for a tumour target, where feasible. However, it was noted that the equivalent tissue histology (e.g. immunohistochemical staining) in in vitro models (mouse tissues or even human organoids) can be used to confirm radiotracer specificity in cases where tumour histology is not feasible. Previous imaging biomarker initiatives—such as The Imaging Biomarker Roadmap—recommend using alternative ‘gold standards’, including biospecimen-derived readouts or whole-tumour 3D analysis to improve the accuracy of imaging–pathology correlation in relevant preclinical and patient studies [31]. Ethical considerations in animal research were emphasised during the consensus discussion, especially in light of the recent FDA Modernization Act 2.0 [59, 60], which permits the use of alternative methods, such as in vitro models [61] and integrated artificial intelligence and computational biology tools [62]. Other preclinical in vivo methods used previously to evaluate radiotracer specificity such as in vivo blocking experiments [63] or patient-derived xenograft studies in which tumour tissues from patients are implanted into immunocompromised or humanised mice [64, 65] were beyond the scope of this consensus discussion, which focused in general on whether preclinical in vivo evidence of radiotracer specificity and stability are required or not for subsequent clinical adoption.

### Preclinical repeatability and reproducibility studies using established or new/novel radiotracers

There was ‘agreement with consensus’ that repeatability studies using an existing radiotracer in a new cancer type are not required to inform on data variability. The panel were also uncertain whether preclinical repeatability studies are needed for new radiotracers. The discussion highlighted that by including groups of test animals and a control group, preclinical studies effectively address variability, thereby reducing the need for repeatability studies within individual animals. Repeatability estimates may also not be feasible in some rodent models due to the rapid growth of tumours, but can be useful in models with slow­growing tumours [31, 66].

Consensus was reached that established radiotracers, repurposed to image new cancer types, do not need to undergo additional preclinical reproducibility studies using different scanners at multiple institutions. It was agreed that preclinical reproducibility studies are not essential for novel radiotracers, although it was noted that this may be dependent on the amount of available preclinical data. It was also acknowledged that the majority of preclinical PET radiotracer studies are single centre as they have the ability to include sufficient animal numbers for appropriate statistical power. Conversely, multicentre approaches are required for clinical studies to reach sufficient patient numbers for statistical significance [26]. Concerns were also raised about variability in PET radiotracer production, stability and availability, which could pose challenges to harmonising preclinical reproducibility studies.

Nevertheless, standardised protocols for preclinical PET/CT imaging, such as those for [^18^F]FDG phantoms, have been shown to improve quantitative accuracy, precision and reproducibility across different institutions and vendors [32]. Efforts to increase awareness of preclinical quality assurance/control guidelines aim to strengthen the reproducibility and reliability of preclinical PET radiotracer data [33, 67]. The discussion also highlighted the precedent set by the MRI community for performing preclinical reproducibility studies across multivendor preclinical scanners at different institutions. For example, test–retest repeatability and cross-site reproducibility phantom studies have been performed to validate measurement of apparent diffusion coefficient imaging biomarker parameters using standardised diffusion- weighted MRI protocols [68, 69] and measurement of water proton longitudinal relaxation rates in small animal MRI scanners [70].

### Preclinical stakeholder engagement

There was ‘agreement without consensus’ (68% agreement) on integrating PPI involvement early in the preclinical study design stage, to obtain a PPI perspective and identify areas of unmet need. It was acknowledged that PPI should be considered from an international perspective as different jurisdictions such as the USA, Europe, Australia and the UK have published their own PPI guidelines, with potentially different strategies [37].

The panel reached consensus that funding bodies should actively support PPI participation and offer guidance to researchers to overcome barriers to incorporating PPI involvement in preclinical research [71]. It was recognised that PPI representatives are already included in animal research ethics committees and are increasingly becoming a prerequisite of funding bodies for preclinical research programmes [71, 72]. For example, funding bodies such as Cancer Research UK, National Institutes for Health and Care Research (NIHR) and UK Research and Innovation have produced guidelines and resources to facilitate researchers in introducing meaningful PPI involvement in their research [73–75]. However, it was recognised that metrics on PPI involvement in preclinical research remain low, warranting greater focus and guidance.

While there was ‘agreement without consensus’ regarding the requirement for a centralised UK preclinical infrastructure, examples like the EuroBioImaging European Research Infrastructure Consortium were noted to show the benefits of shared resources, expertise, training opportunities and data management services [76]. The panel’s uncertainty regarding the necessity of preclinical reproducibility studies and whether funders should incentivise researchers to undertake multicentre preclinical PET radiotracer reproducibility studies influenced their perspective regarding a proposed centralised UK preclinical infrastructure.

There was ‘agreement without consensus’ on the need for an open access database/repository for sharing preclinical radiotracer data to improve transparency and reduce repeating of animal studies unnecessarily. Open access databases have been recommended to facilitate collaboration in preclinical research in personalised medicine [38]. However, the consensus discussion acknowledged that while some funding bodies, such as The National Institutes of Health, Cancer Research UK and NIHR, require preclinical data to be openly accessible in Europe PubMed Central upon final publication, not all publications are open access and some funding bodies do not mandate this. Industry constraints on sharing preclinical or early-phase clinical imaging data were also highlighted due to confidentiality and non-disclosure concerns during product development.

### Clinical studies

Consensus was reached on the need for clinical scanner phantom studies using a novel radiotracer, but ‘uncertainty remained’ for existing radiotracers, applied to image a new cancer type, beyond standard quality control calibration procedures. It was acknowledged that if the new cancer type had a different anatomical distribution, additional phantom studies may be desirable to ensure quantitative accuracy, particularly in characterising the effect of scatter correction on image estimation [77].

For evaluating novel PET radiotracers, there was consensus that clinical dosimetry and/or biodistribution studies are highly appropriate, but unnecessary for established radiotracers with prior relevant data in a different clinical indication. Clinical pharmacokinetic studies were deemed necessary for both novel and existing radiotracers to assess tumour sensitivity and optimise imaging time post injection. The emergence of highly sensitive scanners such as total-body PET, enabling simultaneous imaging of tumour and organ biodistribution and maximal detection of radiation, may enable faster translation of novel radiotracers for human use [44].

There was uncertainty without consensus regarding the need for a clinical comparison study with an established radiotracer such as [^18^F]FDG to validate the diagnostic accuracy of a novel or existing radiotracer for cancer imaging. The discussion highlighted that the need for a comparison study would depend on the objective of the imaging study. For cancer detection studies, comparison with the current standard-of-care radiotracer would be desirable [17, 69–71], although comparison with a different imaging modality could also be considered [72, 73]. For studies investigating whether a tumour expresses a specific receptor, a comparative radiotracer imaging study may not necessarily be required.

Consensus was reached on the appropriateness of conducting clinical repeatability and reproducibility studies to validate new PET radiotracers for cancer diagnosis, prognosis or treatment response detection. However, there was ‘agreement without consensus’ on the need for these studies for existing radiotracers, due to practical constraints such as time and cost, which often deter their inclusion, despite recommendations [31]. Regarding the need for histological analysis of tumours in repeatability/reproducibility studies, there was ‘agreement without consensus’ for novel radiotracers and uncertainty for established radiotracers, with existing histology data in a different cancer type. The discussion emphasised that although histological assessment is desirable, it is not always feasible and therefore, should not be a requirement in repeatability/reproducibility studies.

The panel agreed by consensus that multicentre validation of radiotracers does not require healthy volunteers. Ethical concerns were raised regarding their inclusion in multicentre studies [78], especially considering that cancer patients inherently have both diseased and healthy tissue areas. Recommendations from the Administration of Radioactive Substances Advisory Committee further advocate for limited involvement of healthy volunteers, emphasising age and radiation dose thresholds to ensure safety. Specifically, volunteers taking part in multiple research trials should be over 50 years old, whenever possible, and should not exceed a cumulated annual radiation dose of 10 mSv, from both nuclear imaging and non-nuclear medicine procedures. Specifically, if volunteers below the age of 50 years are required, then specific justification should be included by the clinical radiation expert within their assessment in the clinical trial application [79].

There was strong consensus on the necessity of harmonised study protocols and quality management for multicentre clinical validation studies, with agreement that a centralised PET core lab is recommended to support these studies. However, it was suggested that independent laboratories could potentially perform imaging protocol standardisation, with adequate training to ensure consistent quality standards. Recently, the BNMS have outlined guidelines on quality standards expected for a PET-CT service [80] and The European Association of Nuclear Medicine have provided guidelines on nuclear medicine practices, related to oncology [81].

### Stakeholder involvement in clinical PET radiotracer studies

Consensus was reached on the need for a regulatory framework to standardise PET radiotracer development for cancer imaging. Consensus highlighted that this regulatory framework should require researchers to seek expert statistical advice before embarking on preclinical and clinical validation studies. Additionally, it was agreed that the framework should differentiate between short-term diagnostic and long-term prognostic and therapy response assessment radiotracers, while also providing harmonised guidelines for Research Ethics Committees evaluating the clinical, technical and biological validation evidence.

Consensus highlighted the need for detailed guidance from Health Technology Assessment bodies, such as the National Institute for Health and Care Excellence (NICE), to improve implementation of novel and repurposed radiotracers into clinical practice. However, there was no consensus on health-economic evaluations prior to clinical studies, although the trend leaned towards not required. Uncertainty remained regarding the need for comparative studies with standard-of-care imaging techniques for health-economic evaluation prior to clinical implementation of a new or repurposed radiotracer at a local level (i.e. single site), contingent on the radiotracer’s purpose. However, consensus was reached on their importance at a national level. NHS England currently requires evidence of clinical- and cost-effectiveness prior to commissioning of new radiotracers with marketing authorisation, although unlicensed products may be considered under a ‘specials license’ [32, 33]. NICE also offers guidelines to assess the clinical and cost-effectiveness of radiotracers, compared to established NHS practices [82]. Trials conducted outside the UK must align with NHS practices, focusing on quality-adjusted life years against current standards of care [33]. The BNMS manifesto proposes prioritising clinical need over marketing authorisation, advocating for evidence of incremental benefit, and collaboration between industry, academia and NHS stakeholders for effective radiotracer integration into clinical practice [34].

Consensus highlighted the importance of PPI from the start in defining and prioritising research questions for clinical studies, along with the need for ongoing PPI engagement throughout the research programme. Ensuring diverse PPI representation, encompassing factors such as gender, ethnicity, health literacy, education and socio-economic status is crucial [83], as well as considerations for inclusion of seriously ill and palliative care cancer patients [73]. Recent concerns regarding the potential over-representation of specific demographic groups on PPI panels in cancer research, such as well-educated, proactive females from ethnic majority groups, also warrant attention [84]. Although PPI initiatives are well established in the UK, USA and Australia, expanding such efforts globally is crucial to enhance diversity. Guidance tools, such as the GRIPP2 reporting checklists, aim to assist researchers in improving the design and reporting of PPI practices internationally [85].

Consensus was reached that a centralised UK infrastructure, like NCITA, is necessary to improve the standardisation and reproducibility of imaging biomarkers. Through facilitating collaboration among stakeholders, both nationally and internationally, and improving education and training opportunities, NCITA aims to advance the validation and translation of cancer imaging biomarkers [40]. This aligns with similar successful collaborative initiatives, established internationally, such as the European Society of Radiology European Imaging Biomarkers Alliance [86] and the Radiological Society of North America Quantitative Imaging Biomarkers Alliance [87].

Consensus was agreed on the need for an open access federated repository to facilitate reporting and sharing of clinical imaging data by academia and industry, aligned with the FAIR principles (data must be Findable, Accessible, Interoperable and Reusable) [32]. For example, the National Cancer Institute Cancer Imaging Archive [88] offers an extensive open access clinical imaging data, with associated clinical and genomic information, to foster collaboration and innovation in cancer imaging research. While the consensus panel acknowledged the challenges faced by the industry in sharing early phase clinical data, due to confidentiality concerns, it was agreed that reporting of phase 2 study data and beyond is required. Consensus was reached on the importance of a federated repository in driving the advancement of AI and machine learning methods to improve clinical PET imaging trial workflows, such as imaging data analysis and automated quality control assessments. Additionally, it was recognised that a federated repository would be pivotal in national AI implementation, enabling efficient management of large, well-annotated datasets and facilitating secure and cost-effective data sharing.

Consensus was reached that funders should foster collaborations between UK and international research efforts for PET radiotracer standardisation. Addressing workforce challenges and training needs was also deemed necessary. It was emphasised that urgent action is needed to expand PET scanner availability beyond major tertiary-level cancer centres, improve training for nuclear medicine physicians and radionuclide radiologists, and address healthcare disparities in cancer prognosis, due to limited access to necessary imaging required to deliver targeted therapies [89, 90]. Shortages in the UK radiography workforce [91], alongside global variations in radiography education and access to imaging equipment, underscore the need for standardised education and increased imaging investment worldwide [92], including comprehensive training in multi-modality imaging, in addition to radionuclide imaging [93, 94]. Coordination of workforce training and provision of local and national courses and bespoke training are emphasised by the UK PET-CT advisory board [95] and the UK MR-PET network [96]. The need for comprehensive training to establish a skilled hybrid imaging workforce, aligned with industry needs, is also highlighted by the BNMS UK PET standards, to enhance service quality and patient care outcomes [80].

### Consensus process limitations

As with any consensus process, the results reflect solely the views of the panel and may be prone to biases, including potential pre-selection bias among invited members. To mitigate this, we included a diverse, multi-disciplinary group of stakeholders from leading academic institutions, international companies, hospital trusts, NHS England, funding bodies, regulatory bodies, and PPI advocates. Although the majority of the panel was UK-based, 56% of stakeholders were from outside the NCITA consortium, with representatives from the UK, USA and Australia providing a broad perspective and geographical diversity for consensus discussions on nuclear imaging research practices. Although the integration of radiopharmaceuticals into the NHS provided an example of the regulatory complexities of clinical adoption, the insights derived from our consensus process address challenges across the translational pipeline that are relevant globally. Future consensus studies will seek to expand international perspectives to further mitigate preselection bias.

To reduce bias during the consensus meeting, panellists independently completed the surveys, anonymously rescored their responses and could abstain from answering questions outside their expertise. An independent chair facilitated the meeting, ensuring fair participation, without any individual dominating discussions, and median score distribution graphs were presented to preserve anonymity and reduce bias.

## Conclusions

The field of PET imaging in cancer is advancing rapidly, with the development of a diverse array of radiotracers and highly sensitive imaging techniques. However, bridging the gap between preclinical potential and clinical utility remains challenging. Using a RAM consensus method, we have developed consensus recommendations for improving standardisation of translational methodologies for novel and established radiotracers, applied to image different cancer types. By addressing the challenges and advocating for improvements, such as streamlined regulatory frameworks that do not unnecessarily impede translational progress, improved infrastructure accessibility, stakeholder involvement and workforce training, we aim to accelerate the integration of PET radiotracers into clinical practice for cancer imaging.

## Supplementary Information

Additional file 1: Tables S1–S8. Table S1. NCITA consensus panel members. Table S2. Detailed results for preclinical survey questions on existing radiotracers, novel radiotracers and stakeholder involvement. Table S3. Detailed results for clinical survey questions on existing radiotracers, novel radiotracers and stakeholder involvement. Table S4. First-round survey results (prior to the consensus meeting) for questions on preclinical evaluation methodologies for established and novel radiotracers and stakeholder involvement in the preclinical phase. Table S5. First-round survey results (prior to the consensus meeting) for questions on clinical validation methodologies for established and novel radiotracers and stakeholder involvement in the clinical phase. Table S6. Free-text comments from consensus panel members on preclinical validation requirements for existing or novel radiotracers. Table S7. Free-text comments from consensus panel members on clinical validation requirements for existing or novel radiotracers. Table S8. Free-text comments from consensus panel members on stakeholder involvement in preclinical and clinical radiotracer studies.

## Data Availability

"All data generated or analysed during this study are included in this published article [and its supplementary information files]."

## Abbreviations

PET: Positron emission tomography
RAM: RAND/UCLA Appropriateness Method
PET/CT: Positron emission tomography with computed tomography
PET/MRI: Positron emission tomography with magnetic resonance imaging
[^18^F]FDG): [^18^F]fluoro-2-deoxy-d-glucose
FDA: Food and Drug Administration
PSMA: Prostate-specific membrane antigen
[^18^F]FDOPA: [^18^F]3,4-dihydroxy-6-fluoro-L-phenylalanine
AI: Artificial intelligence tools
NHS: National Health Service
BNMS: British Nuclear Medicine Society
PPI: Patient and public involvement
NCITA: National Cancer Imaging Translational Accelerator
NIHR: National Institutes for Health and Care Research
NICE: National Institute for Health and Care Excellence
