# Supplementary material for: Translation of PET radiotracers for cancer imaging: recommendations from the National Cancer Imaging Translational Accelerator (NCITA) consensus meeting
Source: BMC Med. 2025 Jan 23;23:37. doi: 10.1186/s12916-024-03831-z (PMC11756105; doi:10.1186/s12916-024-03831-z)
Supplement: Supplementary file 1 — Additional file 1: Tables S1–S8. Table S1. NCITA consensus panel members. Table S2. Detailed results for preclinical survey questions on existing radiotracers, novel radiotracers and stakeholder involvement. Table S3. Detailed results for clinical survey questions on existing radiotracers, novel radiotracers and stakeholder involvement. Table S4. First-round survey results (prior to the consensus meeting) for questions on preclinical evaluation methodologies for established and novel radiotracers and stakeholder involvement in the preclinical phase. Table S5. First-round survey results (prior to the consensus meeting) for questions on clinical validation methodologies for established and novel radiotracers and stakeholder involvement in the clinical phase. Table S6. Free-text comments from consensus panel members on preclinical validation requirements for existing or novel radiotracers. Table S7. Free-text comments from consensus panel members on clinical validation requirements for existing or novel radiotracers. Table S8. Free-text comments from consensus panel members on stakeholder involvement in preclinical and clinical radiotracer studies. [file 12916_2024_3831_MOESM1_ESM.docx]

**Table S1. NCITA consensus panel members**

| **Name** | **Institution** | **Expertise/Experience** |
| --- | --- | --- |
| Luigi Aloj | University of Cambridge | Consultant Nuclear Medicine Physician |
| Anna Barnes | King’s Technology Evaluation Centre, King’s College London, and NHS England | Director (KiTEC) / Principal Research Fellow in Healthcare Technologies and former Chief Healthcare Scientist – Southeast Region, Office of the Chief Scientific Officer, NHS-England |
| Phil Blower | King’s College London | Professor of Imaging Chemistry and Head of Department of Imaging, Chemistry and Biology |
| John Braun | Independent | PPI representative |
| Kevin Brindle | University of Cambridge | Professor of Biomedical Magnetic Resonance |
| Craig Buckley | Siemens Healthineers | Head of Research and Innovation GB&I |
| Gary Cook | King’s College London | Professor of Molecular Imaging |
| Penny Hubbard Cristinacce | The University of Manchester | NCITA MRI Core Lab lead |
| Daniel Darian | Siemens Healthineers | Molecular Imaging Collaborations Scientist |
| Paul Evans | GE Healthcare | Head of Global R&D |
| Vicky Goh | King’s College London | Chair of Clinical Cancer Imaging |
| David Grainger | MHRA | Technical Lead: Software, AI and MRI |
| Carol Green | Independent | PPI representative |
| Matt Hall | National Physics Laboratory, UK | Principal Research Scientist |
| Thomas Harding | Prostate Cancer UK | Senior Data & Evidence Officer |
| Geoff Higgins | University of Oxford | Associate Professor in Oncology & Honorary Consultant Clinical Oncologist |
| Catherine Hines | GSK, USA | Executive Director and Head, Clinical Imaging |
| Simon Hollingsworth | AstraZeneca | VP, Global Franchise Head - IO Bispecifics |
| Rowland Illing | University College London and Amazon Web Services | Honorary Clinical Senior Lecturer (UCL) & Director & Chief Medical Officer, International Public Sector Health (AWS) |
| Martin Lee | Independent | PPI representative |
| Hing Leung | University of Glasgow and  CRUK Beatson Institute | Professor of Urology and Surgical Oncology |
| Baptiste Leurent | University College London | Lecturer in Medical Statistics |
| Sue Mallett | University College London | Professor in Diagnostic and Prognostic Medical Statistics |
| Daniel McGowan | Oxford University Hospitals NHS FT & University of Oxford | Head of Education and Research, Consultant Clinical Scientist & Honorary Senior Clinical Research Fellow in Medical Physics |
| Greg Mullen | Theragnostics | Chief Operating Officer – expertise in diagnostic radiopharmaceuticals |
| Radhouene Neji | Siemens Healthineers and King’s College London | Head of Collaborations Magnetic Resonance GB&I (Siemens) and Senior Lecturer in MR Physics (KCL) |
| Tony Ng | King’s College London and GSK | Richard Dimbleby Professor of Cancer Research (KCL) and SVP, Oncology Translational Research (GSK) |
| Natalia Norori | Prostate Cancer UK | Data and Evidence Manager (Epidemiology) |
| James O’Connor | Institute of Cancer Research (ICR), London and The University of Manchester | Professor of Quantitative Biomedical Imaging |
| Nora Pashayan | University College London & University of Cambridge | Professor of Applied Cancer Research |
| Neel Patel | Telix Pharmaceuticals and Oxford University Hospitals FT | VP Clinical Science (Telix), Clinical Lead PET-CT and Nuclear Medicine and Radionuclide Radiologist (OUH) |
| Kieran Prior | Cancer Research UK | Research Programme Manager, Clinical Research |
| Shonit Punwani | University College London | Professor of Magnetic Resonance and Cancer Imaging and Consultant Radiologist |
| Thomas Reiner | Evergreen Theragnostics | Chief Scientific Officer (Evergreen Theragnostics ) – expertise in radioligand therapy |
| Alasdair Taylor | University Hospitals of Morecambe Bay NHS Foundation Trust, Lancaster | Consultant Radiologist & NICE Diagnostic Advisor |
| Jasper van der Aart | GSK, GB | Director, Oncology Clinical Imaging Group |
| Joseph Woollcott | Prostate Cancer UK | Policy & Health Influencing Manager |
| Wai-Lup Wong | NHS England | National Specialty Advisor for Nuclear Medicine |

**Table S1 legend.** This table outlines the affiliations, areas of expertise, and relevant experience of the panel members who contributed to the NCITA consensus process.

**Table S2. Detailed results for preclinical survey questions on existing radiotracers, novel radiotracers and stakeholder involvement**

| **Question** | **Median** | **Median rating appropriateness level** | **Agreement level** | **% median score range distribution** | | | **Abstention**  **N out of 33** |
| --- | --- | --- | --- | --- | --- | --- | --- |
| ***Existing radiotracers – Preclinical*** |  |  |  | **Score 1–3** | **Score 4–6** | **Score 7–9** |  |
| Are **new** *in vitro* studies required for radiotracers used previously clinically in a different indication? | 2 | Not required/ inappropriate | Consensus | 78 | 11 | 11 | 6 |
| Is **new** preclinical evidence from animal studies necessary before starting human studies of the new cancer type? | 3 | Not required/ inappropriate | Consensus | 73 | 23 | 4 | 7 |
| Are **additional** phantom studies required for preclinical scanner calibration and validation? | 8 | Required/ appropriate | Agreement without Consensus | 32 | 8 | 60 | 8 |
| Are dosimetry and**/or** biodistribution studies required in a clinically relevant tumour animal model if human data exists for the radiotracer in a different indication? | 2 | Not required/ inappropriate | Consensus | 88 | 8 | 4 | 7 |
| Are pharmacokinetic studies required in an animal model of the new cancer type to assess **~~sensitivity~~** **specificity** (signal-to-noise and contrast-to-noise ratios) and optimise timing of use of the PET radiotracer? | 2 | Not required/ inappropriate | Consensus | 79 | 17 | 3 | 4 |
| Is histology assessment required to confirm **~~sensitivity~~** **specificity** of the radiotracer in detecting the tumour target? | 7 | Required/ appropriate | Agreement without consensus | 24 | 20 | 56 | 8 |
| **If feasible**, are repeatability (test-retest) studies required to inform on data variability using the same animal? | 2 | Not required/ inappropriate | Consensus | 88 | 12 | 0 | 7 |
| Are reproducibility studies (using different scanners at multiple institutions) required in animals? | 2 | Not required/ inappropriate | Consensus | 89 | 7 | 4 | 5 |

**Table S2 (Continued)**

| **Question** | **Median** | **Median rating appropriateness level** | **Agreement level** | **% median score range distributions** | | | **Abstention**  **N out of 33** |
| --- | --- | --- | --- | --- | --- | --- | --- |
| ***Novel radiotracers – Preclinical*** |  |  |  | **Score 1–3** | **Score 4–6** | **Score 7–9** |  |
| Are *in vitro* cell studies required? | 9 | Required/ appropriate | Consensus | 0 | 8 | 92 | 7 |
| Is preclinical evidence from animal studies necessary? | 9 | Required/ appropriate | Consensus | 4 | 4 | 93 | 5 |
| Are dosimetry and**/or** biodistribution studies required in a clinically relevant tumour animal model? | 9 | Required/ appropriate | Consensus | 0 | 4 | 96 | 7 |
| Are pharmacokinetic studies required to assess **~~sensitivity~~** **specificity** (signal-to-noise and contrast-to-noise ratios) and optimise timing of use of the novel PET radiotracer? | 6 | Uncertain | No consensus | 48 | 7 | 44 | 6 |
| **If feasible**, is histology assessment of the animal tumour required to confirm **~~sensitivity~~ specificity** of the novel radiotracer for tumour detection? | 8 | Required/ appropriate | Consensus | 0 | 0 | 100 | 7 |
| Are repeatability (test-retest) studies required to inform on data variability using the same animal? | 5 | Uncertain | No consensus | 44 | 28 | 28 | 8 |
| Are reproducibility studies (using different scanners at multiple institutions) required in animals? | 2 | Not required / inappropriate | Consensus | 88 | 0 | 12 | 7 |

**Table S2 (Continued)**

| **Question** | **Median** | **Median rating appropriateness level** | **Agreement level** | **% median score range distributions** | | | **Abstention**  **N out of 33** |
| --- | --- | --- | --- | --- | --- | --- | --- |
| ***Stakeholder involvement – Preclinical*** |  |  |  | **Score 1–3** | **Score 4–6** | **Score 7–9** |  |
| Is patient and public involvement (PPI) required during preclinical assessment of PET radiotracers for cancer imaging? | 7 | Required/ appropriate | Consensus | 16 | 16 | 68 | 2 |
| Should funders incentivise PPI involvement during preclinical assessment of radiotracers for cancer imaging (in addition to clinical assessment)? | 7 | Required/ appropriate | Consensus | 13 | 10 | 77 | 2 |
| Should funders provide guidance on PPI involvement during preclinical evaluation of radiotracers for cancer imaging? | 8 | Required/ appropriate | Consensus | 16 | 3 | 81 | 2 |
| Is an open access database required for reporting and sharing of preclinical radiotracer evaluation data from industry and academia to improve transparency of positive and negative results? | 7 | Required / appropriate | Agreement without consensus | 28 | 22 | 50 | 1 |
| Is a centralised UK infrastructure (similar to e.g. Euro Bioimaging) required for preclinical evaluation of radiotracers for cancer imaging to improve data robustness/reproducibility? | 7 | Required/ appropriate | Agreement without consensus | 7 | 33 | 60 | 3 |
| Should funders incentivise preclinical multicentre reproducibility studies to improve preclinical data robustness for successful clinical translation of radiotracers for cancer imaging? | 4 | Uncertain | No consensus | 46 | 25 | 29 | 5 |

**Table S2. Detailed results for preclinical survey questions on existing radiotracers, novel radiotracers and stakeholder involvement.** Table rows coloured green indicate ‘agreement with consensus’ that the survey item was considered ‘required/ appropriate’; rows coloured orange indicate ‘agreement with consensus’ that the survey item was considered ‘not required/ inappropriate’. Table rows coloured blue indicate ‘agreement without consensus’ that the survey item was considered ‘required/ appropriate’, and rows coloured yellow indicate ‘agreement without consensus’ that the survey item was considered ‘not required/ inappropriate’. Table rows coloured grey indicate that ‘uncertainty remained’ regarding the requirement/ appropriateness of a survey item. The number of participants who selected ‘Do Not Know’ for each question is included as ‘Abstention number (N) out of 33’, since 33 panel members out of the original panel of 38 participants completed the second-round rescoring of the consensus survey, three did not participate in the second-round rescoring and two delegated this task to attendees from their institution. Agreed amendments to the wording of the survey questions during the consensus meeting discussion are highlighted in **bold**.

**Table S****3. Detailed results for clinical survey questions on existing radiotracers, novel radiotracers and stakeholder involvement**

| **Question** | **Median** | **Median rating appropriateness level** | **Agreement level** | **% median score range distributions** | | | **Abstention**  **N out of 33** |
| --- | --- | --- | --- | --- | --- | --- | --- |
| ***Existing radiotracers – Clinical*** |  |  |  | **Score 1–3** | **Score 4–6** | **Score 7–9** |  |
| Are **additional** phantom studies required for clinical scanner calibration and validation? | 5 | Uncertain | No consensus | 35 | 23 | 42 | 7 |
| Are clinical dosimetry and**/or** biodistribution studies required if human data exist for the radiotracers in a different indication? | 2 | Not required/ inappropriate | Consensus | 86 | 11 | 4 | 5 |
| Are clinical pharmacokinetic studies required to assess tumour **~~sensitivity~~** **specificity** (signal-to-noise and contrast-to-noise ratios) and optimise timing of use of a PET radiotracer, if human data exist for the radiotracer in a different indication? | 7 | Required/ appropriate | Consensus | 8 | 12 | 81 | 7 |
| **If feasible**, is a comparison study with an established radiotracer such as ^18^F-FDG required for validation of the radiotracer in the new cancer type? | 5 | Uncertain | No consensus | 29 | 46 | 25 | 5 |
| Are repeatability (test-retest) studies required in the same patient for a PET radiotracer used for diagnosis of the new cancer type? | 7 | Required/ appropriate | Agreement without consensus | 7 | 43 | 50 | 5 |
| Are repeatability (test-retest) studies required in the same patient for a radiotracer used to determine patient prognosis or predict clinical outcomes in response to therapy? | 7 | Required/ appropriate | Agreement without consensus | 7 | 36 | 57 | 5 |
| Are reproducibility studies (using different scanners at multiple institutions) required for a PET radiotracer used for diagnosis of the new cancer type? | 8 | Required/ appropriate | Agreement without consensus | 17 | 17 | 67 | 3 |
| Are reproducibility studies (using different scanners at multiple institutions) required for a PET radiotracer used to determine patient prognosis or predict clinical outcomes in response to therapy? | 8 | Required/ appropriate | Agreement without consensus | 13 | 20 | 67 | 3 |
| Should repeatability/reproducibility studies include patient tumour histology analysis? | 5 | Uncertain | No consensus | 46 | 21 | 32 | 5 |

**Table S3 (continued)**

|  | **Median** | **Median rating appropriateness level** | **Agreement level** | **% median score range distributions** | | | **Abstention**  **N out of 33** |
| --- | --- | --- | --- | --- | --- | --- | --- |
| ***Novel radiotracers – Clinical*** |  |  |  | **Score 1–3** | **Score 4–6** | **Score 7–9** |  |
| Are phantom studies required for clinical scanner calibration and validation? | 8 | Required/ appropriate | Consensus | 4 | 11 | 85 | 6 |
| Are clinical dosimetry and biodistribution studies required for novel radiotracers? | 9 | Required/ appropriate | Consensus | 0 | 4 | 96 | 7 |
| Are clinical pharmacokinetic studies required to assess tumour **~~sensitivity~~** **specificity** (signal-to-noise and contrast-to-noise ratios) and/**or** optimise timing of use for novel PET radiotracers? | 9 | Required/ appropriate | Consensus | 0 | 4 | 96 | 6 |
| **If feasible,** is a comparison study with an established radiotracer such as ^18^F-FDG required for validation of the novel radiotracer? | 5 | Uncertain | No consensus | 27 | 40 | 33 | 3 |
| Are repeatability (test-retest) studies required in the same patient for a novel PET radiotracer used for diagnosis of cancer? | 8 | Required/ appropriate | Consensus | 3 | 21 | 76 | 4 |
| Are repeatability (test-retest) studies required in the same patient for a novel PET radiotracer used to determine patient prognosis or predict clinical outcomes in response to therapy? | 8 | Required/ appropriate | Consensus | 3 | 21 | 76 | 4 |
| Are reproducibility studies (using different scanners at multiple institutions) required for a novel radiotracer used for diagnosis of cancer? | 8 | Required/ appropriate | Consensus | 7 | 7 | 87 | 3 |
| Are reproducibility studies required for a novel radiotracer used to determine patient prognosis or predict clinical outcomes in response to therapy? | 8 | Required/ appropriate | Consensus | 3 | 23 | 73 | 3 |
| Should repeatability/reproducibility studies include patient tumour histology analysis? | 7 | Required/ appropriate | Agreement without consensus | 31 | 8 | 62 | 7 |

**Table S3 (continued)**

|  | **Median** | **Median rating appropriateness level** | **Agreement level** | **% median score range distributions** | | | **Abstention**  **N out of 33** |
| --- | --- | --- | --- | --- | --- | --- | --- |
| ***Stakeholder involvement - Clinical*** |  |  |  | **Score 1–3** | **Score 4–6** | **Score 7–9** |  |
| Are healthy volunteers required for multicentre validation of novel or existing PET radiotracers (used previously clinically for a different indication)? | 1 | Not required/ inappropriate | Consensus | 90 | 10 | 0 | 2 |
| Are harmonised study protocols and quality management are required for robust clinical translation of PET radiotracers for cancer imaging? | 9 | Required/ appropriate | Consensus | 0 | 10 | 90 | 4 |
| Should multicentre studies use a centralised PET core lab to harmonise study protocols and standardise quality control of data acquisition, processing and analyses? | 8 | Required/ appropriate | Consensus | 13 | 13 | 73 | 3 |
| Is a regulatory framework required to standardise PET radiotracer development for cancer imaging and accelerate translation into clinical practice? | 7 | Required/ appropriate | Consensus | 6 | 10 | 84 | 2 |
| Should the proposed regulatory framework include a requirement for expert statistical advice for preclinical and clinical evaluation of PET radiotracers for cancer imaging to ensure good statistical design and sample size determination? | 9 | Required/ appropriate | Consensus | 0 | 6 | 94 | 1 |
| Should the proposed regulatory framework distinguish between the validation procedure requirements for short-term diagnostic and long-term prognostic and therapy response assessment radiotracers? | 8 | Required/ appropriate | Consensus | 3 | 22 | 77 | 3 |

**Table S3 (continued)**

|  | **Median** | **Median rating appropriateness level** | **Agreement level** | **% median score range distributions** | | | **Abstention**  **N out of 33** |
| --- | --- | --- | --- | --- | --- | --- | --- |
| ***Stakeholder involvement - Clinical*** |  |  |  | **Score 1–3** | **Score 4–6** | **Score 7–9** |  |
| Should the proposed regulatory framework include harmonised guidelines for Research Ethics Committees (REC) evaluating the clinical, technical and biological validation evidence for cancer imaging radiotracers? | 8 | Required/ appropriate | Consensus | 0 | 10 | 90 | 2 |
| Are guidelines required from Health Technology Assessment bodies such as NICE for successful approval and implementation of novel PET cancer imaging radiotracers into clinical practice? | 8 | Required/ appropriate | Consensus | 0 | 19 | 81 | 2 |
| Are guidelines required from Health Technology Assessment bodies such as NICE for successful approval and implementation of a PET radiotracer (used previously clinically in a different indication) into clinical practice for imaging of a different cancer type? | 8 | Required/ appropriate | Consensus | 7 | 20 | 73 | 3 |
| Should **an initial** health economics evaluation be required prior to clinical validation for novel or existing radiotracers? | 3 | Not required/ inappropriate | Agreement without consensus | 56 | 22 | 22 | 1 |
| Is a comparison study to the SOC imaging technique required for health economics evaluation for implementation of cancer PET imaging radiotracers into clinical practice at a LOCAL level? | 4 | Uncertain | No consensus | 38 | 38 | 24 | 4 |
| Is a comparison study to the standard of care imaging technique required for health economics evaluation for implementation of cancer PET imaging radiotracers into clinical practice at a NATIONAL level? | 8 | Required/ appropriate | Consensus | 10 | 3 | 87 | 3 |
| Should PPI be involved during the early stages of clinical trial development for PET radiotracer validation for cancer imaging? | 8 | Required/ appropriate | Consensus | 10 | 13 | 77 | 2 |

**Table S3 (continued)**

| **Question** | **Median** | **Median rating appropriateness level** | **Agreement level** | **% median score range distribution** | | | **Abstention**  **N out of 33** |
| --- | --- | --- | --- | --- | --- | --- | --- |
| ***Stakeholder involvement - Clinical*** |  |  |  | **Score 1–3** | **Score 4–6** | **Score 7–9** |  |
| Is an open access federated repository required for reporting and sharing of clinical repeatability and reproducibility data by industry & academia to improve successful clinical translation of PET radiotracers for cancer imaging? | 8 | Required/ appropriate | Consensus | 6 | 23 | 71 | 2 |
| Is a federated repository required for AI algorithm development to improve workflows for clinical PET imaging data analysis for successful clinical translation of radiotracers for cancer imaging? | 8 | Required/ appropriate | Consensus | 10 | 19 | 71 | 2 |
| Is a centralised UK infrastructure (e.g. NCITA) required to accelerate successful clinical validation and translation of PET radiotracers for cancer imaging? | 8 | Required/ appropriate | Consensus | 0 | 23 | 77 | 3 |
| Should funders incentivise greater collaboration between UK clinical imaging research and international efforts to improve quantitative PET imaging reproducibility for cancer imaging detection? | 8 | Required/ appropriate | Consensus | 3 | 13 | 83 | 3 |
| Is a gap analysis required to assess workforce issues and training requirements to accelerate the implementation of new PET radiotracer imaging techniques into clinical practice? | 8 | Required/ appropriate | Consensus | 0 | 21 | 79 | 4 |

**Table S3 legend. Detailed results for clinical survey questions on existing radiotracers, novel radiotracers and stakeholder involvement.** Table rows coloured green indicate ‘agreement with consensus’ among the consensus panel that the survey item was ‘required/ appropriate’; rows coloured orange indicate ‘agreement with consensus’ that the survey item was considered ‘not required/ inappropriate’. Table rows coloured blue indicate ‘agreement without consensus’ that the survey item was considered ‘required/ appropriate’, and rows coloured yellow indicate ‘agreement without consensus’ that the survey item was considered ‘not required/ inappropriate’. Table rows coloured grey indicate that ‘uncertainty remained’ regarding the requirement/ appropriateness of a survey item. The number of participants who selected ‘Do Not Know’ for each question is included as ‘Abstention number (N) out of 33’, since 33 panel members out of the original panel of 38 participants completed the second-round rescoring of the consensus survey, three did not participate in the second-round rescoring and two delegated this task to attendees from their institution. Agreed amendments to the wording of the survey questions during the consensus meeting discussion are highlighted in **bold**.

**Table S4. First-round survey results (prior to the consensus meeting) for questions on preclinical evaluation methodologies for established and novel radiotracers and stakeholder involvement in the preclinical phase**

| **Question** | **Median** | **Median rating appropriateness level** | **Agreement level** | **% median score range distribution** | | | **Abstention**  **N out of 32** |
| --- | --- | --- | --- | --- | --- | --- | --- |
| ***Existing radiotracers – Preclinical*** |  |  |  | **Score 1–3** | **Score 4–6** | **Score 7–9** |  |
| Are *in vitro* studies required for radiotracers used previously clinically in a different indication? | 3 | Not required/ inappropriate | Agreement without consensus | 54 | 23 | 23 | 6 |
| Is preclinical evidence from animal studies necessary before starting human studies of the new cancer type? | 3 | Not required/ inappropriate | Agreement without consensus | 58 | 23 | 19 | 6 |
| Are phantom studies required for preclinical scanner calibration and validation? | 8 | Required/ appropriate | Agreement without Consensus | 22 | 26 | 52 | 5 |
| Are dosimetry and biodistribution studies required in a clinically relevant tumour animal model if human data exists for the radiotracer in a different indication? | 2 | Not required/ inappropriate | Agreement without consensus | 67 | 22 | 11 | 5 |
| Are pharmacokinetic studies required in an animal model of the new cancer type to assess sensitivity (signal-to-noise and contrast-to-noise ratios) and optimise timing of use of the PET radiotracer? | 4 | Uncertain | No consensus | 44 | 30 | 26 | 5 |
| Is histology assessment required to confirm sensitivity of the radiotracer in detecting the tumour target? | 6 | Uncertain | No consensus | 19 | 33 | 48 | 5 |
| Are repeatability (test-retest) studies required to inform on data variability using the same animal? | 3 | Not required/ inappropriate | Agreement without consensus | 52 | 33 | 15 | 5 |
| Are reproducibility studies (using different scanners at multiple institutions) required in animals? | 3 | Not required/ inappropriate | Consensus | 73 | 15 | 12 | 6 |

**Table S4 (continued)**

| **Question** | **Median** | **Median rating appropriateness level** | **Agreement level** | **% median score range distributions** | | | **Abstention**  **N out of 32** |
| --- | --- | --- | --- | --- | --- | --- | --- |
| ***Novel radiotracers – Preclinical*** |  |  |  | **Score 1–3** | **Score 4–6** | **Score 7–9** |  |
| Are *in vitro* cell studies required? | 8 | Required/ appropriate | Consensus | 12 | 15 | 73 | 6 |
| Is preclinical evidence from animal studies necessary? | 9 | Required/ appropriate | Consensus | 7 | 11 | 82 | 4 |
| Are dosimetry and**/or** biodistribution studies required in a clinically relevant tumour animal model? | 8 | Required/ appropriate | Consensus | 8 | 23 | 69 | 6 |
| Are pharmacokinetic studies required to assess sensitivity (signal-to-noise and contrast-to-noise ratios) and optimise timing of use of the novel PET radiotracer? | 7 | Required/ appropriate | Agreement without consensus | 12 | 23 | 65 | 6 |
| Is histology assessment of the animal tumour required to confirm sensitivity of the novel radiotracer for tumour detection? | 8 | Required/ appropriate | Consensus | 8 | 15 | 77 | 6 |
| Are repeatability (test-retest) studies required to inform on data variability using the same animal? | 6 | Uncertain | No consensus | 27 | 31 | 42 | 6 |
| Are reproducibility studies (using different scanners at multiple institutions) required in animals? | 4 | Uncertain | No consensus | 46 | 27 | 27 | 6 |

**Table S4 (continued)**

| **Question** | **Median** | **Median rating appropriateness level** | **Agreement level** | **% median score range distributions** | | | **Abstention**  **N out of 32** |
| --- | --- | --- | --- | --- | --- | --- | --- |
| ***Stakeholder Involvement – Preclinical*** |  |  |  | **Score 1–3** | **Score 4–6** | **Score 7–9** |  |
| Is patient and public involvement (PPI) required during preclinical assessment of PET radiotracers for cancer imaging? | 5 | Uncertain | No consensus | 37 | 33 | 30 | 2 |
| Should funders incentivise PPI involvement during preclinical assessment of radiotracers for cancer imaging (in addition to clinical assessment)? | 6 | Uncertain | No consensus | 30 | 30 | 40 | 2 |
| Should funders provide guidance on PPI involvement during preclinical evaluation of radiotracers for cancer imaging? | 7 | Required/ appropriate | Agreement without consensus | 23 | 17 | 60 | 2 |
| Is an open access database required for reporting and sharing of preclinical radiotracer evaluation data from industry and academia to improve transparency of positive and negative results? | 8 | Required / appropriate | Agreement without consensus | 13 | 29 | 58 | 1 |
| Is a centralised UK infrastructure (similar to e.g. Euro Bioimaging) required for preclinical evaluation of radiotracers for cancer imaging to improve data robustness/reproducibility? | 7 | Required/ appropriate | Agreement without consensus | 7 | 37 | 56 | 5 |
| Should funders incentivise preclinical multicentre reproducibility studies to improve preclinical data robustness for successful clinical translation of radiotracers for cancer imaging? | 6 | Uncertain | No consensus | 21 | 31 | 48 | 3 |

**Table S4 legend. First-round survey results (prior to the consensus meeting) for questions on preclinical evaluation methodologies for established and novel radiotracers and stakeholder involvement in the preclinical phase.** Table rows coloured green indicate ‘agreement with consensus’ that the survey item was considered ‘required/ appropriate’; rows coloured orange indicate ‘agreement with consensus’ that the survey item was considered ‘not required/ inappropriate’. Table rows coloured blue indicate ‘agreement without consensus’ that the survey item was considered ‘required/ appropriate’, and rows coloured yellow indicate ‘agreement without consensus’ that the survey item was considered ‘not required/ inappropriate’. Table rows coloured grey indicate that ‘uncertainty remained’ regarding the requirement/ appropriateness of a survey item. The number of participants who selected ‘Do Not Know’ for each question is included as ‘Abstention number (N) out of 32’, as the first-round online survey received 32 out of 34 survey responses (94%) prior to the consensus meeting. This included two joint survey responses from panellists at Prostate Cancer UK (n = 3) and Siemens Healthineers (n = 3).

**Table S5. First-round survey results (prior to the consensus meeting) for questions on clinical validation methodologies for established and novel radiotracers and stakeholder involvement in the clinical phase**

| **Question** | **Median** | **Median rating appropriateness level** | **Agreement level** | **% median score range distributions** | | | **Abstention**  **N out of 32** |
| --- | --- | --- | --- | --- | --- | --- | --- |
| ***Existing radiotracers – Clinical*** |  |  |  | **Score 1–3** | **Score 4–6** | **Score 7–9** |  |
| Are phantom studies required for clinical scanner calibration and validation? | 8 | Required/ appropriate | Agreement without consensus | 27 | 12 | 62 | 6 |
| Are clinical dosimetry and biodistribution studies required if human data exist for the radiotracers in a different indication? | 5 | Uncertain | No consensus | 42 | 35 | 23 | 6 |
| Are clinical pharmacokinetic studies required to assess tumour sensitivity (signal-to-noise and contrast-to-noise ratios) and optimise timing of use of a PET radiotracer, if human data exist for the radiotracer in a different indication? | 7 | Required/ appropriate | Consensus | 12 | 19 | 69 | 6 |
| Is a comparison study with an established radiotracer such as ^18^F-FDG required for validation of the radiotracer in the new cancer type? | 5 | Uncertain | No consensus | 31 | 24 | 45 | 3 |
| Are repeatability (test-retest) studies required in the same patient for a PET radiotracer used for diagnosis of the new cancer type? | 6 | Uncertain | No consensus | 7 | 44 | 48 | 5 |
| Are repeatability (test-retest) studies required in the same patient for a radiotracer used to determine patient prognosis or predict clinical outcomes in response to therapy? | 6 | Uncertain | No consensus | 19 | 37 | 44 | 5 |
| Are reproducibility studies (using different scanners at multiple institutions) required for a PET radiotracer used for diagnosis of the new cancer type? | 7 | Required/ appropriate | Agreement without consensus | 21 | 21 | 59 | 3 |
| Are reproducibility studies (using different scanners at multiple institutions) required for a PET radiotracer used to determine patient prognosis or predict clinical outcomes in response to therapy? | 8 | Required/ appropriate | Agreement without consensus | 14 | 21 | 66 | 3 |
| Should repeatability/reproducibility studies include patient tumour histology analysis? | 7 | Required/ appropriate | Agreement without consensus | 27 | 23 | 50 | 6 |

**Table S5 (continued)**

| **Question** | **Median** | **Median rating appropriateness level** | **Agreement level** | **% median score range distributions** | | | **Abstention**  **N out of 32** |
| --- | --- | --- | --- | --- | --- | --- | --- |
| ***Novel radiotracers – Clinical*** |  |  |  | **Score 1–3** | **Score 4–6** | **Score 7–9** |  |
| Are phantom studies required for clinical scanner calibration and validation? | 9 | Required/ appropriate | Consensus | 4 | 8 | 88 | 6 |
| Are clinical dosimetry and biodistribution studies required for novel radiotracers? | 9 | Required/ appropriate | Consensus | 0 | 0 | 100 | 6 |
| Are clinical pharmacokinetic studies required to assess tumour sensitivity (signal-to-noise and contrast-to-noise ratios) and optimise timing of use for novel PET radiotracers? | 9 | Required/ appropriate | Consensus | 0 | 0 | 100 | 7 |
| Is a comparison study with an established radiotracer such as ^18^F-FDG required for validation of the novel radiotracer? | 7 | Required/ appropriate | Agreement without consensus | 21 | 25 | 54 | 4 |
| Are repeatability (test-retest) studies required in the same patient for a novel PET radiotracer used for diagnosis of cancer? | 8 | Required/ appropriate | Agreement without consensus | 4 | 31 | 65 | 6 |
| Are repeatability (test-retest) studies required in the same patient for a novel PET radiotracer used to determine patient prognosis or predict clinical outcomes in response to therapy? | 8 | Required/ appropriate | Consensus | 4 | 22 | 74 | 5 |
| Are reproducibility studies (using different scanners at multiple institutions) required for a novel radiotracer used for diagnosis of cancer? | 8 | Required/ appropriate | Consensus | 4 | 18 | 79 | 4 |
| Are reproducibility studies required for a novel radiotracer used to determine patient prognosis or predict clinical outcomes in response to therapy? | 8 | Required/ appropriate | Consensus | 4 | 19 | 78 | 5 |
| Should repeatability/reproducibility studies include patient tumour histology analysis? | 7 | Required/ appropriate | Agreement without consensus | 15 | 19 | 65 | 6 |

**Table S5 (continued)**

| **Question** | **Median** | **Median rating appropriateness level** | **Agreement level** | **% median score range distributions** | | | **Abstention**  **N out of 32** |
| --- | --- | --- | --- | --- | --- | --- | --- |
| ***Stakeholder involvement - Clinical*** |  |  |  | **Score 1–3** | **Score 4–6** | **Score 7–9** |  |
| Are healthy volunteers required for multicentre validation of novel or existing PET radiotracers (used previously clinically for a different indication)? | 4 | Uncertain | No consensus | 50 | 32 | 18 | 4 |
| Are harmonised study protocols and quality management are required for robust clinical translation of PET radiotracers for cancer imaging? | 9 | Required/ appropriate | Consensus | 0 | 10 | 90 | 2 |
| Should multicentre studies use a centralised PET core lab to harmonise study protocols and standardise quality control of data acquisition, processing and analyses? | 8 | Required/ appropriate | Agreement without consensus | 10 | 31 | 59 | 3 |
| Is a regulatory framework required to standardise PET radiotracer development for cancer imaging and accelerate translation into clinical practice? | 7 | Required/ appropriate | Consensus | 6 | 13 | 81 | 1 |
| Should the proposed regulatory framework include a requirement for expert statistical advice for preclinical and clinical evaluation of PET radiotracers for cancer imaging to ensure good statistical design and sample size determination? | 8 | Required/ appropriate | Consensus | 0 | 10 | 90 | 1 |
| Should the proposed regulatory framework distinguish between the validation procedure requirements for short-term diagnostic and long-term prognostic and therapy response assessment radiotracers? | 7 | Required/ appropriate | Consensus | 3 | 28 | 69 | 3 |

**Table S5 (continued)**

| **Question** | **Median** | **Median rating appropriateness level** | **Agreement level** | **% median score range distributions** | | | **Abstention**  **N out of 32** |
| --- | --- | --- | --- | --- | --- | --- | --- |
| ***Stakeholder involvement - Clinical*** |  |  |  | **Score 1–3** | **Score 4–6** | **Score 7–9** |  |
| Should the proposed regulatory framework include harmonised guidelines for Research Ethics Committees (REC) evaluating the clinical, technical and biological validation evidence for cancer imaging radiotracers? | 8 | Required/ appropriate | Consensus | 3 | 19 | 77 | 1 |
| Are guidelines required from Health Technology Assessment bodies such as NICE for successful approval and implementation of novel PET cancer imaging radiotracers into clinical practice? | 8 | Required/ appropriate | Consensus | 3 | 23 | 74 | 1 |
| re guidelines required from Health Technology Assessment bodies such as NICE for successful approval and implementation of a PET radiotracer (used previously clinically in a different indication) into clinical practice for imaging of a different cancer type? | 8 | Required/ appropriate | Agreement without consensus | 10 | 24 | 66 | 3 |
| Should **an initial** health economics evaluation be required prior to clinical validation for novel or existing radiotracers? | 6 | Uncertain | No consensus | 32 | 19 | 48 | 1 |
| Is a comparison study to the SOC imaging technique required for health economics evaluation for implementation of cancer PET imaging radiotracers into clinical practice at a LOCAL level? | 6 | Uncertain | No consensus | 14 | 45 | 41 | 3 |
| Is a comparison study to the standard of care imaging technique required for health economics evaluation for implementation of cancer PET imaging radiotracers into clinical practice at a NATIONAL level? | 8 | Required/ appropriate | Consensus | 3 | 23 | 73 | 2 |

**Table S5 (continued)**

| **Question** | **Median** | **Median rating appropriateness level** | **Agreement level** | **% median score range distributions** | | | **Abstention**  **N out of 32** |
| --- | --- | --- | --- | --- | --- | --- | --- |
| ***Stakeholder involvement - Clinical*** |  |  |  | **Score 1–3** | **Score 4–6** | **Score 7–9** |  |
| Should PPI be involved during the early stages of clinical trial development for PET radiotracer validation for cancer imaging? | 8 | Required/ appropriate | Consensus | 10 | 14 | 76 | 3 |
| Is an open access federated repository required for reporting and sharing of clinical repeatability and reproducibility data by industry & academia to improve successful clinical translation of PET radiotracers for cancer imaging? | 8 | Required/ appropriate | Consensus | 3 | 27 | 70 | 2 |
| Is a federated repository required for AI algorithm development to improve workflows for clinical PET imaging data analysis for successful clinical translation of radiotracers for cancer imaging? | 7 | Required/ appropriate | Agreement without consensus | 13 | 30 | 57 | 2 |
| Is a centralised UK infrastructure (e.g. NCITA) required to accelerate successful clinical validation and translation of PET radiotracers for cancer imaging? | 8 | Required/ appropriate | Consensus | 0 | 25 | 75 | 4 |
| Should funders incentivise greater collaboration between UK clinical imaging research and international efforts to improve quantitative PET imaging reproducibility for cancer imaging detection? | 8 | Required/ appropriate | Consensus | 3 | 28 | 69 | 3 |
| Is a gap analysis required to assess workforce issues and training requirements to accelerate the implementation of new PET radiotracer imaging techniques into clinical practice? | 8 | Required/ appropriate | Consensus | 0 | 31 | 69 | 3 |

**Table S5 legend. First-round survey results (prior to the consensus meeting) for questions on clinical validation methodologies for established and novel radiotracers and stakeholder involvement in the clinical phase.** Table rows coloured green indicate ‘agreement with consensus’ that the survey item was considered ‘required/ appropriate’; rows coloured blue indicate ‘agreement without consensus’ that the survey item was considered ‘required/ appropriate’; rows coloured grey indicate that ‘uncertainty remained’ regarding the requirement/ appropriateness of a survey item. The number of participants who selected ‘Do Not Know’ for each question is included as ‘Abstention number (N) out of 32’, as the first-round online survey received 32 out of 34 survey responses (94%) prior to the consensus meeting. This included two joint survey responses from panellists at Prostate Cancer UK (n = 3) and Siemens Healthineers (n = 3).

**Table S6. Free-text comments from consensus panel members on preclinical validation requirements for existing or novel radiotracers.**

| **Free-text (Survey section 1)**  **Existing radiotracers – Preclinical** | **Free-text (Survey section 2)**  **Novel radiotracers - Preclinical** |
| --- | --- |
| - The way the questions are posed is rather equivocal. I'm not sure a one size fits all approach works. It should be considered on a case by case basis and depending on what existing evidence there is that the particular pathway/target is present in the unproven indication. | - Again, case by case and depending on how effective/sensitive/target to background features of the compound. If results are clear-cut there may be no need to do validation at multiple sites/scanners. |
| - Preclinical animal studies of tracers in the contexts described above are unsound ethically and economically since there is nothing to stop evaluation directly in humans. The important thing is to have sufficient mechanistic evaluation to understand what the scan means at the molecular level. Histological studies associated with first use in the new cancer are the most appropriate and reliable. general conclusions such as "this tracer binds selectively to this receptor" are best identified in vitro. | - Need animal studies for toxicity data and to test pharmacokinetics and cell type distribution, as there is no prior human use data |
| - Need only studies to establish that tracer is relevant in the new cancer cell type. Pharmacokinetic studies from previous human studies should be best starter for dose, better than an animal study. Calibration is always needed. Reproducibility and repeatability on humans surpass those on animals, so if there is human data on reproducibility (doses and different scanners), it would be only relevant to know this data for that cancer or a similar tissue that takes up tracer at a similar amount | - Q11 and Q12, I put 7 as presumably we can get as much information as possible from animal models before inconveniencing patients. - Q14 if image guided histology, then would score 9. |
| - For Q6, I put 5 as don't know. If it was image guided histology assessment, then I would answer 9. | - It is more important to perform animal studies on a novel radiotracer due to safety considerations. However, assessment of how similar the novel radiotracer is to a previously validated radiotracer may affect what is necessary. |
| - Considering the implementation of the FDA Modernization Act 2.0 on Dec 29, 2022 that “allows for alternatives to animal testing for purposes of drug and biological product applications”, we should question whether animal studies are required to conduct imaging research. |  |

**Table S6.** **Free-text comments from consensus panel members on preclinical validation requirements for existing or novel radiotracers.** Consensus panel members had the option to provide additional comments in free-text boxes at the end of survey section questions addressing preclinical validation requirements for existing (Table S2, section 1) or novel (Table S3, section 1) radiotracers.

**Table S7. Free-text comments from consensus panel members on clinical validation requirements for existing or novel radiotracers**

| **Free-text (Survey section 4)**  **Existing radiotracers - Clinical** | **Free-text (Survey section 5)**  **Novel radiotracers - Clinical** |
| --- | --- |
| - I have assumed for Q33 you mean analysis of histology rather than +ve histology before trial entry. | - Again, uncertain if this is a one size fits all solution for many of these items given the hypothetical premise. |
| - Repeatability and reproducibility studies are required when radiotracer being used to generate biomarker measurements, regardless of purpose of biomarker i.e. diagnosis or prognosis. The difference between those studies is how long between the biomarker measurement at baseline, and the outcome event that the biomarker is related to i.e. both types of study have same needs for reproducibility and repeatability of biomarker baseline measurements. I put 8 not 9, as I can imagine some situations where you don't need a study e.g. very similar tracer. | - These questions do not address the main issues around study design which influence clinical adoption. Clinical adoption is becoming more influenced by whether the decisions made using the tracer influence therapeutic outcome, not these technical issues. |
| - For Q27 I put 5 for don't know because not all cancer cells are alike. For Q33, I put 7 because if the biopsy is not image guided it could be wrong. | - For Q40, I have said not required as the changes to be measured are of the order of 30-50% so a few % either way are not a problem. - Q43 would be 9 if it was PET image guided biopsy. |
|  | - It is important to perform safety checks and biological and technical validation on a novel radiotracer. Comparison with other established biomarkers and histology is biomarker-dependent. |

**Table S7. Free-text comments from consensus panel members on clinical validation requirements for existing or novel radiotracers.** Consensus panel members had the option to provide additional comments in free-text boxes at the end of survey section questions addressing clinical validation requirements for existing (Table S2, section 2) or novel (Table S3, section 2) radiotracers.

**Table S8. Free-text comments from consensus panel members on stakeholder involvement in preclinical and clinical radiotracer studies**

| **Free-text (Survey section 3)**  **Stakeholder involvement - Preclinical** | **Free-text (Survey section 6)**  **Stakeholder involvement - Clinical** |
| --- | --- |
| - I am always wary about what 'open' means when connected with data, so Q21 - Q23 I need more information (I'm a lay person). | - A dramatic step change in provision of PET scanners and training of radiologists outside the major UK tertiary-level cancer centres will be required before meaningful progress can be made in implementing new strategies. Current provision of 1 PET scanner serving a resident population of 2.6 million cannot hope to meet the challenges ahead and if this gap is not addressed, patients face a potential postcode lottery in cancer prognosis due to inability to access the imaging required to deliver targeted therapies. |
| - I think all of these things are desirable but not deal breakers. There is no way to prove undoubtedly with preclinical imaging that a particular test that works in a mouse will work in a human. | - Questions about regulatory framework seem essential to me so I don't understand why there would even be a question. I need more explanation. |
| - I'm not clear if PPI is required for animal studies, but it is required for human studies. | - Q48 - target product profiles (TPP, as MHRA can help organise) are helpful to accelerate pathway, as they give clear expectations for a successful product. - Q50 - if you are using validation to mean evaluation that determines clinical utility in final population, then need a separate TPP for diagnosis and prognosis as these are separate "use cases" i.e. separate clinical uses and so separate TPPs. - Q51 - I'm not sure what you are proposing for DMC guidance. Most use DAMOCLES which has to cover lots of different situations. The study itself and TPP may define when the study needs to be closed for futility or AEs. |

**Table S8 (Continued)**

| **Free-text (Survey section 3)**  **Stakeholder involvement - Preclinical** | **Free-text (Survey section 6)**  **Stakeholder involvement - Clinical** |
| --- | --- |
| - Open sharing of data and analysis may allow fewer preclinical studies to be performed, potentially reducing animal use. | - Q56 need a comparison study, and modelling of impact at multiple sites if the clinical pathway is different at different hospitals. Don't need HE at every local centre. - Q58 is a good idea, but unclear how you can enforce to industry (or even academics). It might be that there is a registry of what has been tried, even if results are not included? - Q59 AI repository sounds like a waste of money (for government) but the AI companies will love shifting their costs to the taxpayer! I think that before tax payer funds, there should be some proper use case made, and also a very good robust example of AI being useful. With CAD (AI by a slightly different name) we don't have good examples of usefulness that would justify a repository. - Q62 - remember to include statistical support needs in gap analysis, including for the sample sizes in all the studies, as well as for the design aspects |
|  | - Use of a centralised PET Core Lab is useful but there is a need for capacity to be available. |

**Table S8. Free-text comments from consensus panel members on stakeholder involvement in preclinical and clinical radiotracer studies.** Consensus panel members had the option to provide additional comments in free-text boxes at the end of survey section questions addressing stakeholder involvement in preclinical (Table S2, section 3) and clinical (Table S3, section 3) radiotracer studies.
